# Case Report: Low-Dose Apatinib in the Treatment of Intrahepatic Biliary Cystadenoma With Recurrence and Malignant Transformation

**DOI:** 10.3389/fonc.2021.676092

**Published:** 2021-06-28

**Authors:** Yongguang Yang, Weiheng Mai, Weifeng Chen, Chao Yang, Mingyi Li, Lijuan Liu

**Affiliations:** ^1^ Department of Hepatobiliary Surgery, The Affiliated Hospital of Guangdong Medical University, Zhanjiang, China; ^2^ Department of Ultrasound Diagnostics, The Affiliated Hospital of Guangdong Medical University, Zhanjiang, China

**Keywords:** apatinib, intrahepatic biliary cystadenoma, intrahepatic biliary cystadenocarcinoma, targeted therapy, intrahepatic bile duct cystic tumor

## Abstract

Apatinib is a new oral tyrosine kinase inhibitor that targets vascular endothelial growth factor receptor-2. It has been proven effective in treating multiple solid tumors. Herein, we report the case of a 67-year-old Chinese patient who was diagnosed with recurrent and malignant transformation of intrahepatic biliary cystadenoma. After multidisciplinary team discussion, the team considered that the remaining liver volume was insufficient for surgical resection. The patient refused chemotherapy and radiotherapy and was willing to take apatinib. Initially, the patient experienced severe tongue ulcers and difficulty eating. The dose of apatinib was then adjusted to 250 mg/day. To date, he has been taking apatinib for 48 months. Regular re-examination showed that the tumor had significantly decreased in size. On January 16, 2021, a CT scan revealed a tumor diameter of 4.5 cm. In our case, the patient achieved partial response and progression-free survival(PFS) of 48.0 months. During treatment, the patient’s appetite and mental state were expected. The treatment did not induce hypertension, fatigue, hand-foot syndrome, or liver and kidney damage. Apatinib may be an option for the treatment of advanced intrahepatic biliary cystadenocarcinoma. Its toxicity is controllable and tolerable. The exact curative effect still needs to be evaluated in more cases.

## Background

Apatinib (Hengrui Pharmaceutical Co., Ltd., Shanghai, China.) is a multi-target, small-molecule tyrosine kinase inhibitor that can selectively inhibit the tyrosine kinase activity of vascular endothelial growth factor receptor-2 (VEGFR-2) and inhibit tumor neovascularization, thus suppressing tumor growth. Clinical observations have shown that apatinib has a specific anti-tumor effect in various tumors (1–3). However, there are no reports on the effectiveness and safety of apatinib in treating patients with intrahepatic biliary cystadenocarcinoma (IBCA). Here, we report, for the first time, the case of a 67-year-old male patient with IBCA who showed a satisfactory response to low-dose apatinib in our department.

## Case presentation

**Figure 1:**
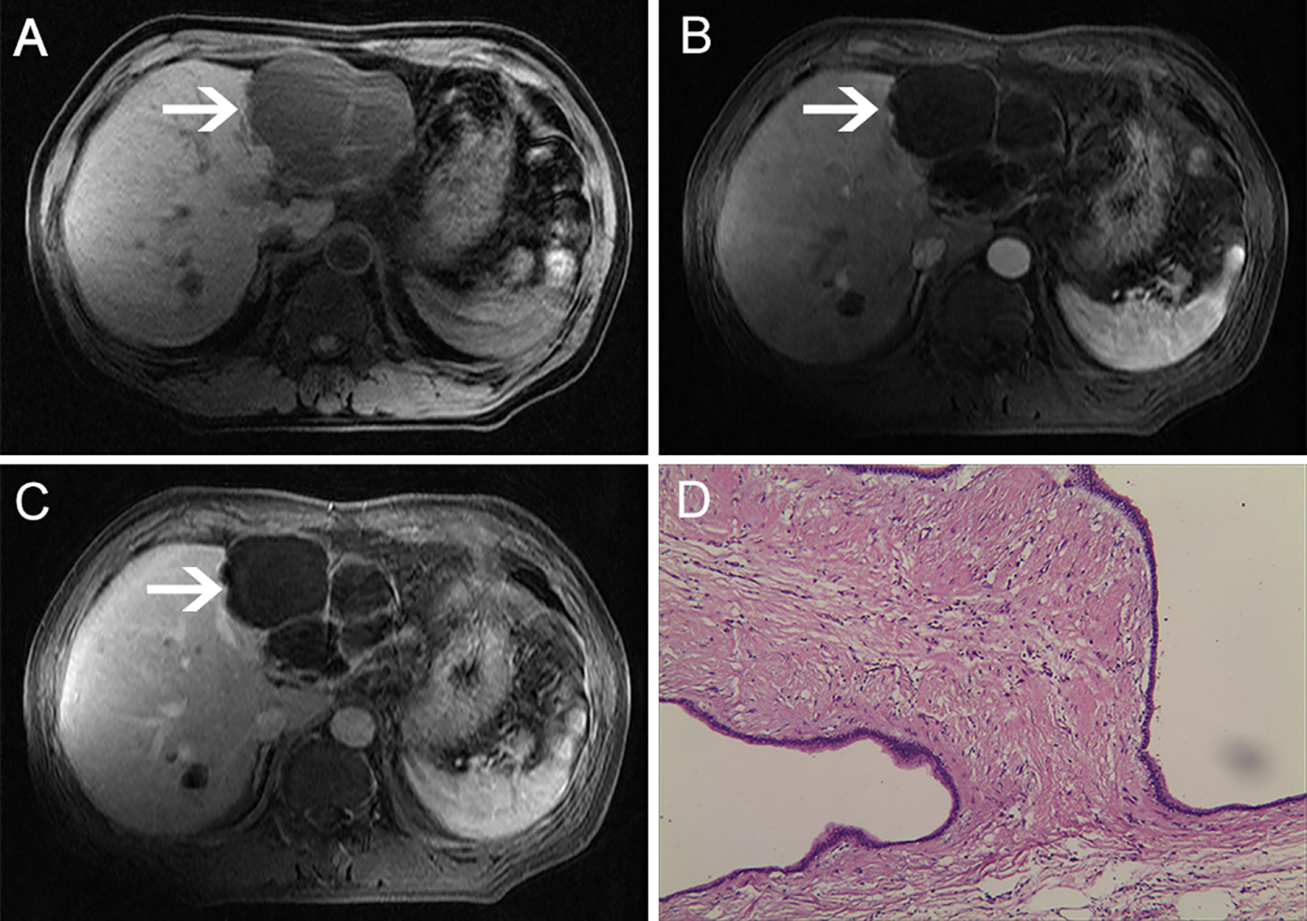
MRI examination. The lesion showed low signals with multiple rooms on T1-weighted images. **(A)** Contrast-enhanced T1-weighted images showed no lesion enhancement: **(B)** arterial phase; **(C)** venous phase. **(D)** Microscopically, multiple cysts were observed. The inner wall of the cyst was covered with columnar mucous epithelium, the cytoplasm was slightly eosinophilic, and the nucleus was located at the base (HE ×200).

**Figure 2:**
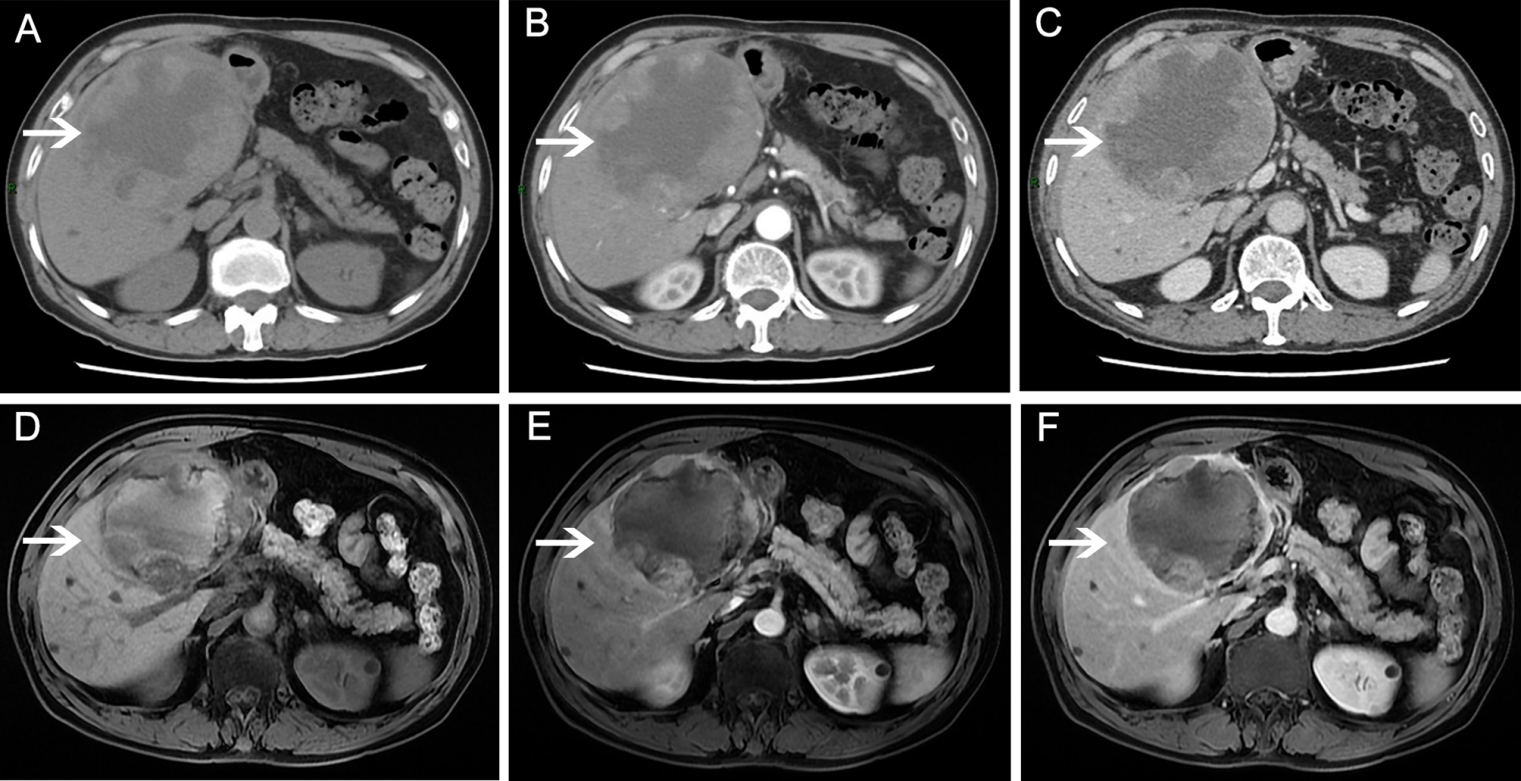
Abdominal magnetic resonance imaging (MRI)/computed tomography (CT) scans before and after apatinib therapy. Before apatinib treatment, a CT scan showed a mass with cystic and solid masses. **(A)** plain scan; **(B)** arterial phase; **(C)** venous phase. After 3 months of apatinib treatment, MRI revealed that the tumor mass decreased significantly. **(D)** T1-weighted image; **(E)** arterial phase; **(F)** venous phase.

**Figure 3:**
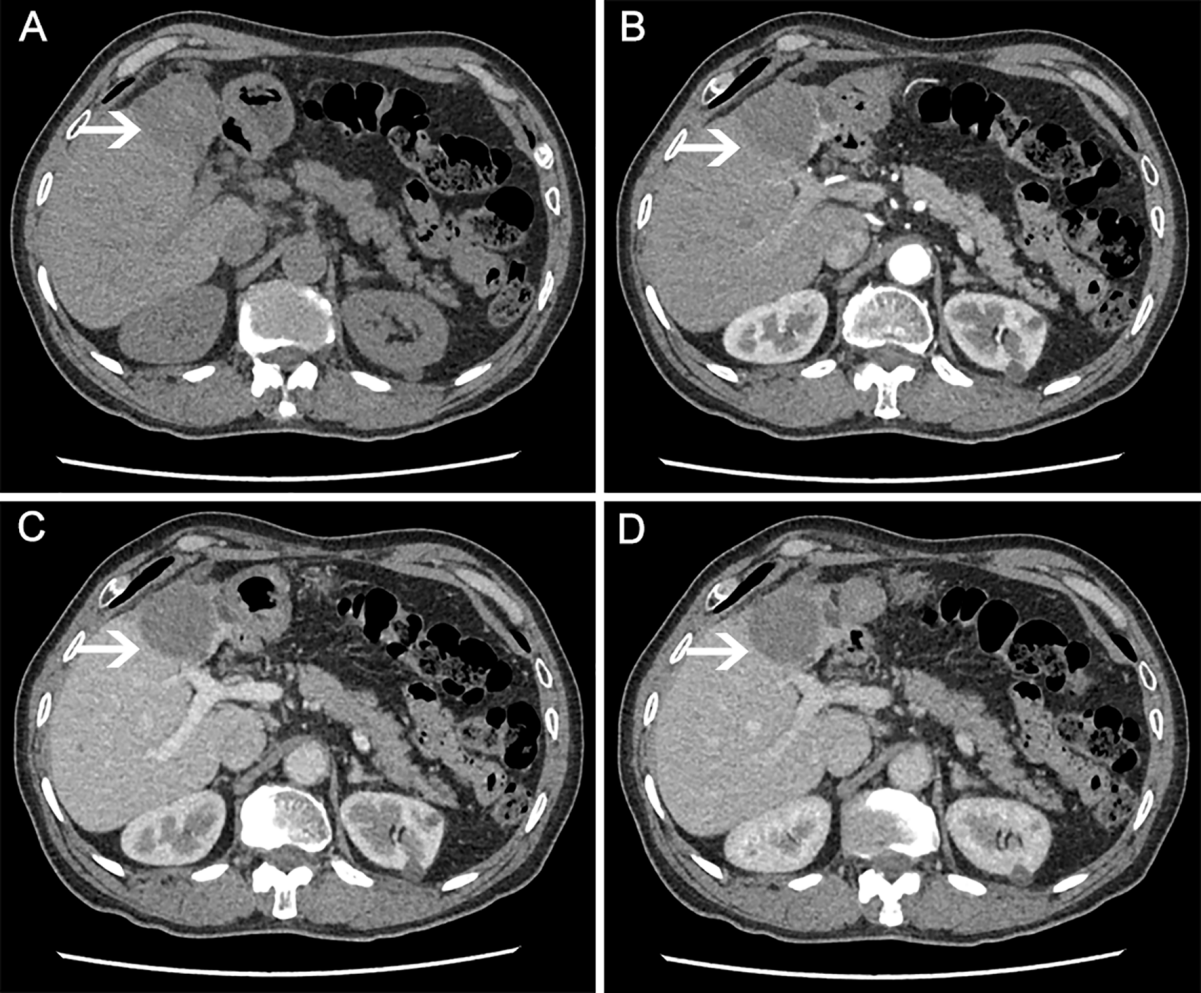
CT scan (on January 21, 2021) showing liver mass after 48 months of apatinib treatment. **(A)** plain scan; **(B)** arterial phase; **(C)** venous phase; **(D)** equilibrium phase.

On December 10, 2016, a 67-year-old man was admitted to our hospital to further evaluate a liver mass discovered during routine postoperative examination in a local hospital. The patient had no clinical discomfort. Past History: On December 2, 2011, the patient underwent a left lateral hepatectomy because of abdominal magnetic resonance imaging (MRI) showing a multilocular cystic mass in the left lateral lobe of the liver (**Figures 1A–C**). Postoperative pathology confirmed intrahepatic biliary cystadenoma (IBC) (**Figure 1D**). No recurrence was found during regular follow-up 3 years after the operation. On December 15, 2016, a computed tomography (CT) scan of the abdomen in our hospital revealed a 16×12 cm mass with cystic and solid masses, accompanied by mixed density and papillary excrescences. After the contrast agent’s infusion, the lesions showed inhomogeneous enhancement, with a low-density necrotic area in the arterial phase and decreased enhancement in the venous phase (**Figures 2A–C**). Serum CA19-9 level was 8.4 U/mL(normal range: 0-27 U/mL), Serum CEA level was 1.67 ng/mL (normal range: 0-5 ng/mL). After multidisciplinary team discussion, we diagnosed the patient with recurrent and malignant transformation of IBC. We assessed that the patient’s remaining liver volume was not sufficient to undergo liver resection. The patient resisted radiotherapy, chemotherapy, and interventional therapy and only agreed to take apatinib treatment. After signing an informed consent form, the patient started taking apatinib 500 mg/day on February 3, 2017. After taking medicine for 10 days, the patient experienced severe tongue ulcers and difficulty in eating. Therefore, the patient stopped taking apatinib and was treated symptomatically. After 15 days, the discomfort symptoms were relieved, and the dose of apatinib was adjusted to 250 mg/day. MRI (on May 25, 2017) showed that the tumor had shrunk to 12×9 cm, which indicated that the patient achieved stable disease (**Figures 2D–F**). On July 15, 2020, the patient underwent tension-free repair of the right inguinal hernia in our department and recovered well after the operation. On January 16, 2021, a CT scan showed that the tumor had a diameter of 4.5 cm. No noticeable enhancement was observed (**Figures 3A–D**). During the follow-up period, no other organs or lymph node metastasis were observed found. The serum tumor marker levels of CEA, CA199, were within the normal range. To date, this patient has been taking apatinib for 48 months. The patient achieved a partial response (PR) according to the Response Evaluation Criteria in Solid Tumours (RECIST) 1.1 standard, and PFS of 48.0 months was achieved. No adverse events, such as hypertension, hand-foot syndrome, liver and kidney function damage, thyroid dysfunction, and digestive tract discomfort, were detected during treatment. The low-dose apatinib was well-tolerated by the patient. The patient agreed to continue taking the medication and refused radical surgical resection.

## Discussion

Intrahepatic bile duct cystic tumor (IBCT) is a rare hepatic cystic tumor, and its aetiology remains unknown. It accounts for 5% of all intrahepatic cystic diseases (4). Due to progress in understanding IBCT, there is an increasing number of related reports. Its actual incidence should be higher, as it is often misdiagnosed as a simple hepatic cyst or other cystic lesions (5). Most of the lesions are single, mostly in the intrahepatic bile duct and rarely in the extrahepatic bile duct; lesions in the gallbladder are rare (6), and most cysts are different IBCT. IBCT is classified into two histological types: IBC and IBCA (7, 8). IBC is a precancerous lesion with a malignant transformation rate of up to 30% (5).

IBCA is a multilocular liver malignant tumor originating from the IBC and is composed of mucous-secreting epithelial cells (7, 9). Previous studies have reported that this disease often occurs in middle-aged women (10). Recent studies have found that the disease is more common in men aged between 55 and 60 years, and the incidence is higher in Asian people (9). Devaney et al. (7) proposed two subtypes of IBCA: (1) malignant transformation caused by IBC with or without ovarian stroma, which is the most common subtype in women, and (2) malignant transformation caused by intrahepatic bile duct or bile duct malformation, which is the subtype that is more common in men.

The clinical manifestations of IBCA and IBC are very similar, making diagnosis difficult. If an ultrasound shows that the intracapsular sound transmission is poor, the cyst fluid is thick, the intracapsular septum is thickened, and the mural nodule is larger than 1.0 cm with coarse calcification; cystadenocarcinoma may also be indicated (11). Choi et al. suggested that CT scan and MRI were excellent in facilitating the differential diagnosis of the two diseases (12). Calcification in the cyst wall, solid masses in the cyst (wall nodules), and thickening of the cyst wall or septum have a tendency to be diagnosed as cystadenocarcinoma. Enhanced CT scans can identify, with greater clarity, the intracapsular septum and potential solid masses in the cyst; however, MRI can determine, with greater clarity, whether the intracapsular septum is connected to the bile duct, thus improving diagnosis rate and assisting in making the operation plan (13). For imaging diagnosis, with respect to suspected IBC or IBCA, there is a risk of transmission, in addition to the limited diagnostic value of the fluid composition characteristics of the needle tract. Therefore, invasive examination or percutaneous fine-needle aspiration cytology is not recommended (8).

Surgical resection is the first choice for treating IBCT because of the difficulty in diagnosing IBCA before surgery and the high malignant transformation rate (14). The possibility of IBC or IBCA should be considered for patients with atypical hepatic cystic lesions. For any suspected hepatobiliary cystadenoma, standard lobectomy should be performed as far as possible to ensure complete resection of the cystic wall and to reduce the recurrence rate (15). During an operation of a patient with IBCA, destroying the integrity of the tumor should be avoided, the scope of resection should be at least approximately 2 cm from the edge of the cyst, and the cyst fluid should be prevented from leaking into the abdominal cavity, which will cause extensive metastasis while ensuring an excellent margin to achieve good prognosis (16). Atypical IBC can easily be misdiagnosed as a hepatic cyst. When thickening of the cyst wall occurs, yellow vegetation on the cyst wall, turbid flocculent or viscous fluid accompanied by bile-like fluid or dark red bloody fluid, or different shapes of polycystic liver cyst fluid may be observed during an operation; in such cases, multi-point sampling is recommended to avoid the omission of bad lesions and the possibility of secondary operation (17). In this case, the patient should undergo resection after drug treatment. Unfortunately, the patient refused to receive surgery or pathological puncture for accurate pathological result.

IBCA is not sensitive to radiotherapy or chemotherapy, and advanced patients lack effective treatment. Radiotherapy and chemotherapy are suitable for patients with advanced tumors, unresectable operations, or late remission of symptoms (18, 19). In a study by Zhao et al. (20), patients with advanced IBCA were treated with docetaxel combined with cisplatin and hyperthermia, and after six cycles of treatment, the lesions did not progress. There have been no reports of targeted therapy for IBCA.

Tumour angiogenesis is a critical step in tumor growth and metastasis. It plays a vital role in providing oxygen, nutrition, and growth factors for tumors; therefore, anti-angiogenesis agents can treat solid tumors (21). Apatinib is a small-molecule tyrosine kinase inhibitor that highly selectively binds to and strongly inhibits VEGFR-2, decreasing VEGF-mediated endothelial cell migration, proliferation, and tumor microvascular formation (22). The China Food and Drug Administration recommended apatinib as a third-line treatment for advanced gastric and gastroesophageal junction adenocarcinoma in 2015. Besides, apatinib shows excellent potential in various solid tumors, such as non-small cell lung cancer (23), breast cancer (24), hepatocellular carcinoma (3), pancreatic cancer (25), and ovarian cancer (26). Adverse reactions to apatinib include hypertension, hand-foot syndrome, albuminuria, fatigue, anorexia, and elevated transaminase level; most of these adverse events are of grade1-2, which can be relieved by drug withdrawal or reduction (3, 27). We report a patient with advanced IBCA who showed a very encouraging response to apatinib. The patient achieved a PR, and a PFS of 48.0 months was achieved, which shows that angiogenesis also plays a vital role in cholangiocarcinoma’s occurrence and development. In addition, apatinib was well-tolerated by the patient, which further proved its safety and suitability. It is suitable as the choice of long-term maintenance treatment. We also think that adverse effects were controlled by dose reduction, or interruption, and symptomatic treatment. Through this patient’s treatment process, combined with relevant literature reports at home and abroad, we get the following enlightenment: We administer drug dose according to the patient’s body weight and age. In the beginning, a medium dose is recommended, which can be increased if the patient is well tolerated; On the contrary, if the adverse reaction is severe, the dose can be reduced to maintain the long-term curative effect.

## Conclusions

IBCA is a rare malignant tumor of the liver. IBCA is not sensitive to radiotherapy and chemotherapy, and patients with advanced IBCA lack effective treatment. We think that apatinib may be a new option for the Treatment of IBCA. Its efficacy and safety need to be confirmed by further study..

## Data Availability Statement

The original contributions presented in the study are included in the article/**Supplementary Material**. Further inquiries can be directed to the corresponding authors.

## Ethics Statement

The studies involving human participants were reviewed and approved by The Ethics Committee of Affiliated Hospital of Guangdong Medical University. The patients/participants provided their written informed consent to participate in this study. Written informed consent was obtained from the individual(s) for the publication of any potentially identifiable images or data included in this article.

## Author Contributions

YY and ML performed the surgery. WC and WM wrote the original draft. CY collected and arranged imaging and pathological data. LL designed the study and revised the manuscript. All authors contributed to the article and approved the submitted version.

## Funding

This study was supported by the National Science Foundation of Guangdong Province (Grant no: 2018A030307076).

## Conflict of Interest

The authors declare that the research was conducted in the absence of any commercial or financial relationships that could be construed as a potential conflict of interest.
