# Supplementary figures and images for: Case Report: Low-Dose Apatinib in the Treatment of Intrahepatic Biliary Cystadenoma With Recurrence and Malignant Transformation
Source: Front Oncol. 2021 Jun 28;11:676092. doi: 10.3389/fonc.2021.676092 (PMC8273731; doi:10.3389/fonc.2021.676092)

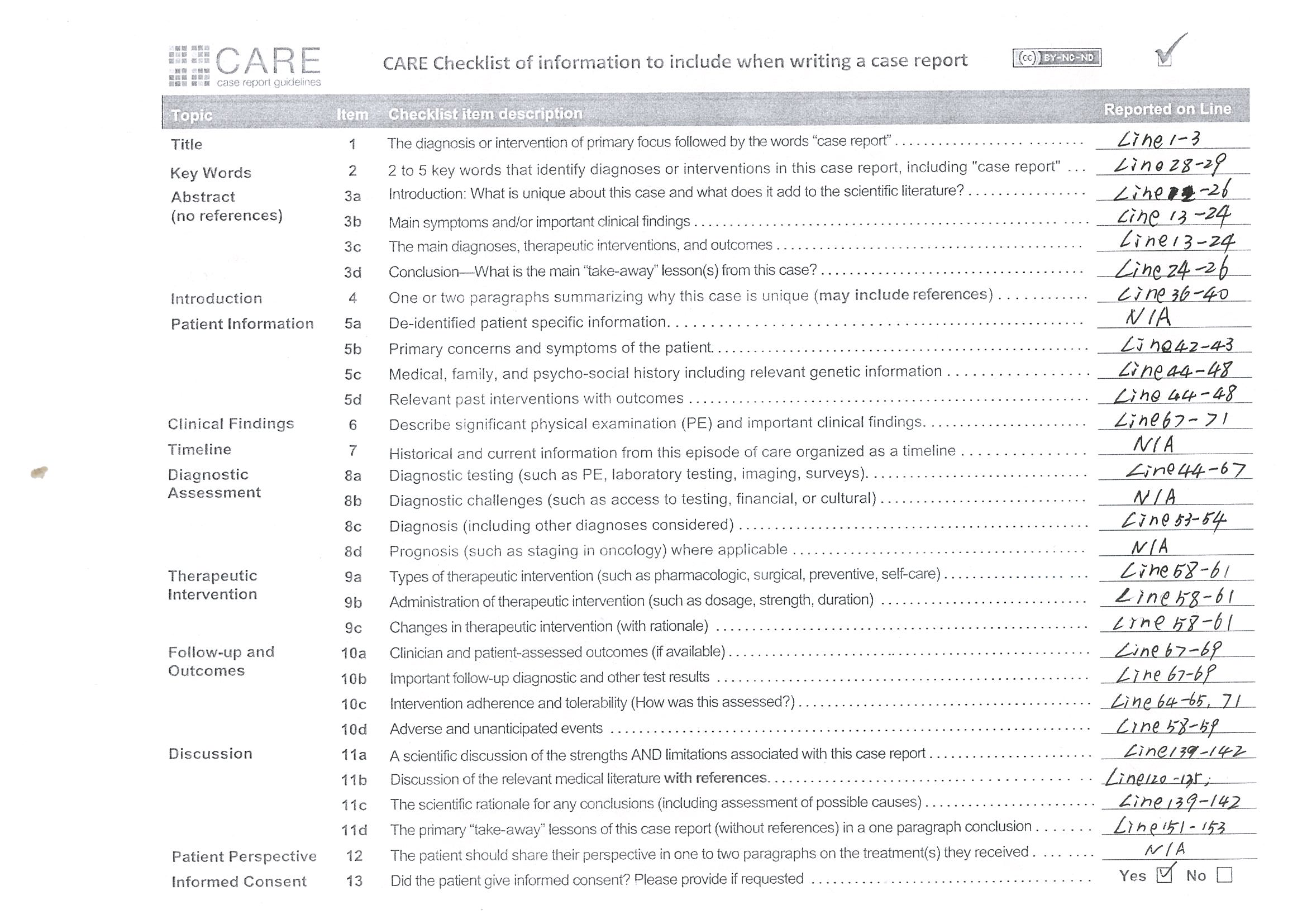

Supplement: Supplementary file 1 [file Image_1.tif]
